# Genome and gene alterations by insertions and deletions in the evolution of human and chimpanzee chromosome 22

**DOI:** 10.1186/1471-2164-10-51

**Published:** 2009-01-26

**Authors:** Natalia Volfovsky, Taras K Oleksyk, Kristine C Cruz, Ann L Truelove, Robert M Stephens, Michael W Smith

**Affiliations:** 1 Advanced Biomedical Computing Center, Advanced Technology Program, SAIC-Frederick, National Cancer Institute at Frederick, Frederick, MD 21702, USA; 2 Laboratory of Genomic Diversity, National Cancer Institute at Frederick, Frederick, MD 21702, USA; 3 Basic Research Program, SAIC-Frederick, National Cancer Institute at Frederick, Frederick, MD 21702, USA; 4 Department of Biology, University of Puerto Rico, Mayagüez, PR 00681, Puerto Rico

## Abstract

**Background:**

Understanding structure and function of human genome requires knowledge of genomes of our closest living relatives, the primates. Nucleotide insertions and deletions (indels) play a significant role in differentiation that underlies phenotypic differences between humans and chimpanzees. In this study, we evaluated distribution, evolutionary history, and function of indels found by comparing syntenic regions of the human and chimpanzee genomes.

**Results:**

Specifically, we identified 6,279 indels of 10 bp or greater in a ~33 Mb alignment between human and chimpanzee chromosome 22. After the exclusion of those in repetitive DNA, 1,429 or 23% of indels still remained. This group was characterized according to the local or genome-wide repetitive nature, size, location relative to genes, and other genomic features. We defined three major classes of these indels, using local structure analysis: (i) those indels found uniquely without additional copies of indel sequence in the surrounding (10 Kb) region, (ii) those with at least one exact copy found nearby, and (iii) those with similar but not identical copies found locally. Among these classes, we encountered a high number of exactly repeated indel sequences, most likely due to recent duplications. Many of these indels (683 of 1,429) were in proximity of known human genes. Coding sequences and splice sites contained significantly fewer of these indels than expected from random expectations, suggesting that selection is a factor in limiting their persistence. A subset of indels from coding regions was experimentally validated and their impacts were predicted based on direct sequencing in several human populations as well as chimpanzees, bonobos, gorillas, and two subspecies of orangutans.

**Conclusion:**

Our analysis demonstrates that while indels are distributed essentially randomly in intergenic and intronic genomic regions, they are significantly under-represented in coding sequences. There are substantial differences in representation of indel classes among genomic elements, most likely caused by differences in their evolutionary histories. Using local sequence context, we predicted origins and phylogenetic relationships of gene-impacting indels in primate species. These results suggest that genome plasticity is a major force behind speciation events separating the great ape lineages.

## Background

In the past decade, the focus of genomic diversity studies mainly included single nucleotide polymorphisms (SNPs) and short tandem repeats (STRs) in humans as well as in other organisms. More recently, the International HapMap project directed efforts toward understanding haplotype structure in human populations and helped to form our perception of genomic diversity based on SNPs [1-4]. At the same time, Olson and Varki [5] argued that understanding our own genome would not be complete without the evolutionary perspective, and requires knowledge of the genomes of our closest primate relatives [5,6]. Previously, direct comparisons of human and chimpanzee sequences have used either a single chromosome [7] or the entire genomes [8]. Resulting extensive sequence datasets opened a possibility for carefully examining alternative sources of genomic variation, such as insertions and deletions (indels).

Since humans diverged from a common ancestor with chimpanzees approximately 5 million years ago [16], understanding genome differences between these two lineages is critically important for defining our own species. Several studies that examined chimpanzee and human genomes comparatively, located and characterised sequence differences, including single-base pair indels, monomeric and multi-base pair extensions (repeats), indels with random DNA sequences, and transposon insertions [5,10,15]; and more are currently under way. Initially, differences between humans and chimpanzees were estimated at 1% [7,17,18], but later this number was refined to 1.2% [8]. Several studies pointed out that the number of differences is much higher when indels (insertions and deletions) are included in the comparison [19-21], and the total divergence may be as high as 6.5% [19]. Removing repeats and low-complexity DNA reduces this calculation to 2.4% [19], doubling the original estimates.

Indels, fragments missing in sequence comparisons between individuals or closely related species, are plentiful across genomes [9,10]. Only a small fraction of indels occurs within coding sequences; it seems that these may play a key role in primate evolution [19,22]. While most indels have no adaptive value, some are known to alter important functions, and many are known to be involved in disease phenotypes [11-14]. It has been noted that while the human genome might contain as many as 1.6–2.5 million indel polymorphisms, efforts directed toward the discovery of this type of genomic variants are still significantly less intensive than efforts involved in the SNP discovery [10]. Many indel polymorphisms can still be discovered and classified in comparative genomic studies.

As the number of described indels accumulates, several mechanisms have been proposed to explain their source and existence. The origin of individual indels seems to depend on their size, sequence context as well as other factors [15,23,24]. For example, it appears that many recent short insertions in the human genome originated as tandem duplications, while smaller indels (<5 bp) were generated either by unequal crossing over or by replication slippage [23]. In addition, a non-homologous end joining mechanism initiated by a double-strand breakage [15] has also been proposed as a main mechanism of indel generation for a wide range of sizes [24].

In this study, we report on those indels (10 bp or larger) that cannot be simply explained by short tandem repeats and/or other repetitive DNA occurrence. We obtained a set of indels by comparing homologous sections of human and chimpanzee chromosome 22 (following the orthologous numbering nomenclature [8,25]), and characterized it relative to the local, chromosomal, genomic, and gene-specific sequence contexts. We divided our data into three groups (referred to as "core classes") according to presence, sequence identity, and relative locations of additional copies of indel sequence in the neighboring (± 5 kb) region. We also considered the observed indels in their genomic context and further divided the data into three groups (referred to as "genome classes") based on the presence of copies locally on chromosome 22 or elsewhere in the human genome. The presence of indels in coding sequences of genes and other genomic elements was examined relative to the random expectation using a tenfold chromosome-wide resampling approach. Finally, we examined predicted transcripts for their impacts on peptide sequence to confirm genes where an insertion or deletion can change the amino acid sequence or alternate splice products that differentiate between the two species. Indels that impacted genes by altering coding sequences and splice sites were further characterized. Gene impacts were considered both computationally, by looking at the resulting amino acid sequence, and by direct sequencing, in comparison among five human populations and with five closely related primate species.

## Results

### Identification and analysis of indels

We identified 6,278 indels (≥ 10 bp) in a comparison between 33 Mb of syntenic regions from human and chimpanzee chromosome 22, using human genome as a reference (Fig. S1(see Additional file 1)). Human sequence extended from 15.4 to 49.3 Mb while the chimpanzee sequence covered a region from 15.4 to 50.0 Mb (Table 1). Most of these variants were found around simple sequence repeats (2,572), but similar numbers were located in and around the known repetitive DNA sequences (2,277) and were filtered out (Table 1). For simplicity, throughout this paper we refer to the data set containing the remaining 1,429 repeat-filtered sequences as the "observed" dataset. In addition, we also generated a tenfold random resampled dataset of fragments of human chromosome 22 with distribution of lengths identical to those of the previously described observed dataset (see Materials and Methods, Fig. S1(see Additional file 1)). We will refer to this artificially generated data set and the "indels" it contains as "resampled," and consider it as the baseline expectation in our subsequent statistical analysis.

**Table 1:** Indels Observed in the Human and Chimpanzee Chromosome 22 Comparison

**Type**	**Total bp**	**bp %**	**# Events**	**# Events %**
**STRs**	**614,359**	**1.8**	**2572**	**41.0**
**Known Repetitive DNA**	**640,133**	**1.9**	**2277**	**36.2**
SINE/Alu	355,124	(1.07)	1593	
Line/L1	138,880	(0.4)	247	
SINE/Mir	18,767	(0.05)	69	
LTR/ERV1	60,003	(0.18)	232	
Other	67,359	(0.2)	136	
**Indels**	**82,661**	**0.25**	**1429**	**22.8**
Insertion	34,868	(0.11)	746	
Deletion	47,793	(0.14)	683	
**TOTAL**	**1,760,410**	**5.3**	**6278**	**100**

A total of 6,278 indels of size ≥ 10 bp were seen in comparison of human and chimpanzee chromosomes 22. Most of these were either in Short Tandem Repeats (STRs) or Known Repetitive DNAs, where the major types are shown. The remaining indels (0.25%) were classified as insertions or deletions relative to the human sequence. Another 0.25% of the indels were 2–9 bp (19,932, totalling 76,486 bp). In addition, 346,771 Single Nucleotide Differences were seen in the UCSC alignment totalling 1.0% of the bases compared.

### Identification and characterization of genomic context and core indel types

**Figure 1:**
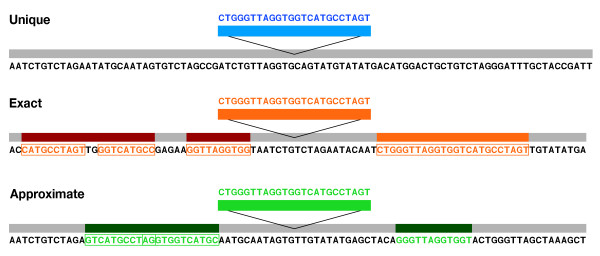
**Classification of Indels into Core Types, Based on the Flanking Sequence**. The indels are classified into 3 core types based on their similarity to the sequences in the 5 Kb flanking regions. The unique type is formed by the indels with no similarity to the flanking regions. The indels with at least one exact copy of indel sequence in the flanking regions define the exact type; and the approximate type includes indels with only partial (sub-repeats of indel sequence) or complex (combination of indels sub-repeats) copies of indel in the flanking region. Sub-repeats (length of ≥ 10 bp) are shown in frames, colors are used to designate between different core types: unique (blue), exact (red) and approximate (green).

Indels in the observed dataset were classified according to their local or genome-wide repetitive nature. Both datasets (observed and resampled) were divided into three core classes based on local structure analysis: (i) those found uniquely in the 10 Kb indel-harbouring region were marked as "unique" (31%), (ii) those with at least one exact copy found locally were categorized as "exact" (12%), and (iii) those with similar but not identical copies of the core indel sequence found locally were labelled as "approximate"(58%) (Fig. 1, Table S1). In addition, we divided the observed dataset into three genome classes according to the presence and location of additional copies of sequences in the human genome (Table S1, Fig. S2 (see Additional file 1)). A group of indels whose exact sequences (including the indel and ± 18 bp of the flanking sequence) were unique to their genome location were the most common ("genome-unique," 92.3%), followed by the group of indels with copies present elsewhere, but exclusively on chromosome 22 ("chromosome-unique," 7.2%). There was also a small fraction of indels whose copies were also identified on other chromosomes of the human genome, outside of chromosome 22 ("chromosome-multiple," 0.5%). Our subsequent analysis was based on comparisons among genome or core classes of intels, as well as on contrasting between the observed data and the baseline provided by the tenfold resampled dataset.

**Figure 2:**
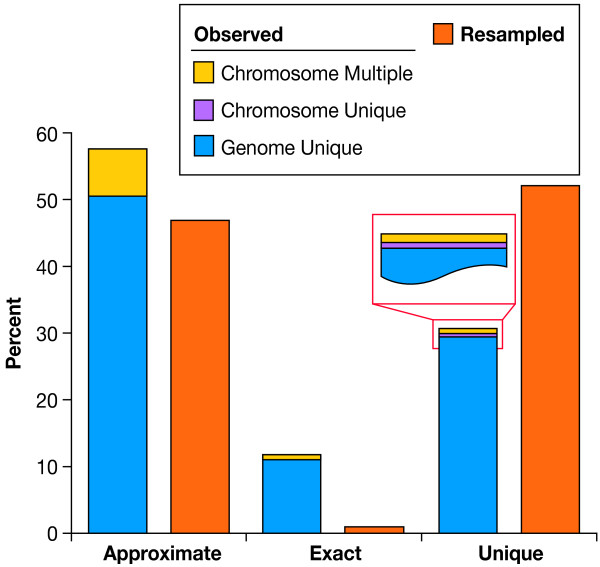
**Distribution of Indels Among Different Genome and Core Indel Classes**. Frequency of the three core indel classes: approximate, exact, and unique, are contrasted in the observed and resampled datasets (orange bars) There is an excess of observed approximate and exact indels, and a shortage of unique indels compared to the expected values for chromosome 22 (LR χ^2^, d.f. = 2, χ^2 ^= 916, p < 0.0001). Colours within the bars representing observed data indicate the relative frequency of the three genome classes (chromosome-multiple, chromosome-unique, and genome-unique). The distribution of core indel types among the genome classes is not random with majority represented by genome-unique indels (LR χ^2^, d.f. = 4, χ^2 ^= 23.28, p = 0.0001, Table S2.1A) (see Additional file 1)).

Indels from the three core classes (approximate, exact, or unique) were not represented equally among the three genome classes (genome-unique, chromosome-unique, or chromosome-multiple) (LR χ^2 ^= 23.28, 4 d.f., p = 0.0001, Table S2.1A (see Additional file 1)). We identified a significant excess of approximate and exact indels at the expense of unique indels in the observed dataset (Likelihood Ratio (LR) χ^2 ^= 916, 2 d.f., P < 0.0001; Table S1(see Additional file 1) and Fig. 2). The majority of genome-unique indels had copies locally and were classified as approximate core indels (56.3%) reflecting their locally repetitive nature (Table S1(see Additional file 1)). Most chromosome-unique indels were also found in the approximate core class (75.7%). At the same time, the unique core indels (those with no copies in the 10 Kb flanking sequence) were better represented among the genome-unique (no copies elsewhere in the genome) (31.7%) compared to the chromosome-unique indels (copies unique to the chromosome) (14.6%) (Table S1, Fig. S2(see Additional file 1)).

### Characterization of core indel classes among and within genic features

**Figure 3:**
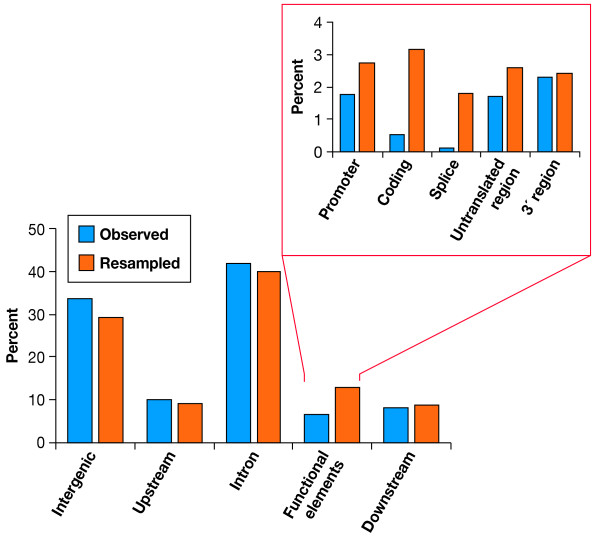
**Distribution of Indels Classified by Their Location Relative to Gene Elements**. Indels were distributed unequally across the genome with most of them present within the introns and the intergenic regions (lower panel). There were significantly fewer observed indels within functional elements group than expected (LR χ^2^, d.f. = 4, χ^2 ^= 27.63, p < 0.0001). The chart in the upper panel represents distribution of indels within the functional element category classified further by specific functional region. Promoter, coding regions and splice sites contain many fewer observed indels than expected (LR χ^2^, d.f. = 4, χ^2 ^= 46.82, p < 0.0001).

The locations of 1,429 observed and 14,290 expected indels were assessed in relation to RefSeq genes annotated on chromosome 22 and assigned into five gene element categories: (1) intergenic sequence, sequences located (2) upstream and (3) downstream of known genes, (4) functional gene elements, and (5) introns (Fig. 3, bottom panel). The observed frequency distribution of indels among these five gene element categories was not random (LR χ^2 ^= 27.63, 4 d.f., p < 0.0001; Fig. 3, bottom). Indels were less frequent immediately upstream (10.1%) and downstream (8.6%) of the genes, and in functional regions of genes (6.5%; Fig. 3, bottom). Most of the observed indels were found either in introns and intergenic regions (33.5 and 41.8%; respectively, Fig. 3). The overall distribution of the three indel core classes among the five categories also differed from the distribution of the resampled dataset (p = 0.02; Table S3), largely due to fewer approximate indels located within genes than expected (6.5% vs. 13.5%, LR χ^2 ^= 31.6, 4 d.f., p < 0.0001, Table S3(see Additional file 1)).

Indels located within genes were divided into five more categories according to the type of functional gene elements as located in: (a) promoters, (b) coding sequence, (c) splicing sites, (d) 3' gene flanking regions, and (e) untranslated regions (UTRs) (Fig. 3, top panel). Three of the five gene functional elements – promoter sites, splice sites, and coding regions – contained fewer observed indels than expected (LR χ^2 ^= 46.82, d.f. = 4, P < 0.0001; Table S4 (see Additional file 1)). At the same time, slightly more indels than expected were found within the UTRs and 3' flanking regions (Fig. 3, top panel).

The three core classes of indels differed in their proximity to genes estimated as a distance to the boundary of the nearest exon (Fig. S3(see Additional file 1)): approximate indels were found furthest away from genes ($\bar{\text{x}}$ = 8,886 bp), followed by the unique ($\bar{\text{x}}$ = 5,792 bp), and exact ($\bar{\text{x}}$ = 6,877 bp) categories (p = 0.02, Table S2.1B (see Additional file 1)). The observed distribution of distances between indels and genes differed among indel core classes, and between the observed and the resampled data (p < 0.0001, Table S2.1B (see Additional file 1)). In the observed dataset, unique indels were located closer to coding sequences than approximate indels (6,877 vs. 8,886 bp on average, p < 0.0001; Table S2A (see Additional file 1)). Those unique indels found within introns were, on average, closer to exons than expected (1,175 vs. 1,651 bp on average, p = 0.004; Table S2.1B (see Additional file 1)). Additionally, both approximate and unique indels, within the functional gene elements were on average twice as far from exons than expected (146 vs. 341 bp and 165 vs. 387 bp, respectively (Table S2.1B (see Additional file 1)), and overall, observed indels were found further away from gene elements than expected (GLM, p < 0.0001).

### Length and distance to the nearest exon differences among the indel types

**Figure 4:**
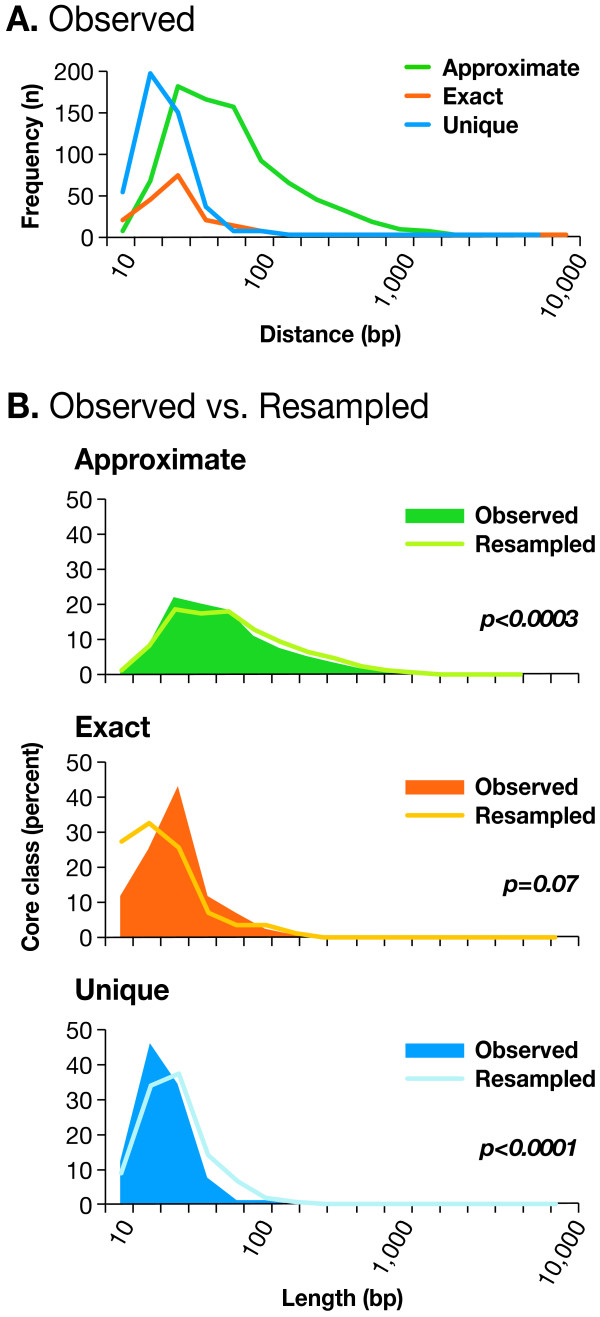
**Distribution of Indel Length Among the Three Core Classes**. (A) Approximate indels have the largest length, followed by exact, and then unique (Table S2.1A, p < .0001). (B) Approximate and unique indels are shorter than expected (Table S1.1A, p < .0001 (see Additional File 1)). Distribution of exact indels in both here and in Fig. S3 appears jagged due to the lower sample size (n = 168) in this class compared to the other two: approximate and unique.

A comparison of indel lengths (Fig. 4 and Table S2.1(see Additional file 1)). A and among the three core classes and across gene elements was performed. Approximate indels, making up the largest core class, were shorter in length in the observed compared to the resampled dataset (47 vs. 52 bp on average, p = 0.0003; Table S2.1A (see Additional file 1)). Unique and exact indels were similar in size, and both contained indels that were on average smaller than their counterparts in the approximate class (p < 0.0001, Table S2.1A and Fig. S1(see Additional file 1)). Observed indels located within genes were the shortest (26 bp), shorter than that expected from the resampled distribution (34 bp on average, p < 0.0001; Table S2.1A (see Additional file 1)). Approximate indels showed the greatest difference in size within gene elements (p < 0.0001, Table S2.1A (see Additional file 1)), while their presence in other categories, such as introns and intergenic elements, had no effect on length (p = 0.2–0.7, Table S2.1A (see Additional file 1)). Unique indels were shorter than expected overall, but the most significant differences between observed and expected length were found among those unique indels located outside genes, rather than within gene elements (p = 0.007–0.009, Table S2.1A (see Additional file 1)).

### Evaluation of the observed indel impacts on gene structure

**Figure 5:**
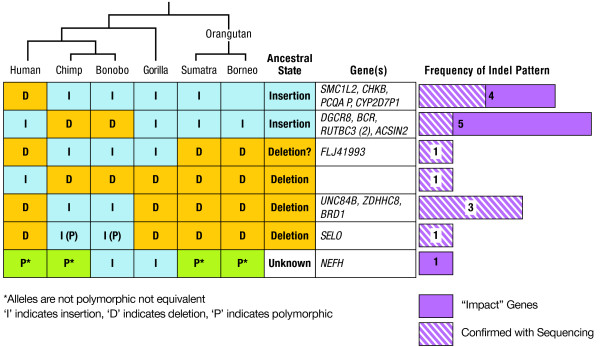
**Laboratory results of the insertions/deletions that are predicted to have an effect on coding regions of genes**. 23 indels found in coding exons or in splice site regions of the human REFseq gene. The inferred ancestral state of each indel was based on whether a species has the insertion or deletion of that locus and the species' position on the phylogenetic tree.

Using a comparative approach, we reconstructed indel fragments within the human sequence that potentially impacted genes, and evaluated them either by deleting insertions or by adding deletion sequences (relative to the chimpanzee). Among the 23 indels identified in coding exons or in splice sites, 10 had potential impact on protein products: seven resulted in truncated proteins, two caused short insertions of amino acids, and one resulted in an amino acid substitution in eight impacted genes: *CELSR1, FLJ41993, FLJ44385, NEFH, RUTBC3, SMC1L2, TCF20*, and *UNC84B5 *(Table 2). The inferred ancestral state of each indel was based on whether the species interrogated in the laboratory had an insertion or a deletion relative to the known primate phylogenies (Fig. 5). The other 13 predicted indels observed in *ACSIN, BCR*, *BRD1*, *CHKB*, *CYP2D7P1*, *DGCR8*, *PCQAP*, *PRODH*, *RUTBC3*, *SBF1*, *SELO*, and *ZDHHC8 *genes, had no effect on the predicted coding sequence (not shown).

Along with the computational evaluation, the gene impacting indels were also examined by direct PCR and sequencing, using primers from conserved flanking sequences. The presence of an insertion or deletion was then examined by PCR amplification in 189 samples from five human populations (Asians, American Indians, African Americans, Africans, and European Americans), as well as 26 chimpanzees, 11 bonobos, nine gorillas and 10 individuals from two subspecies of orangutans (five samples each; see Materials and Methods). Products were sized on agarose gels to identify polymorphisms and species differences in these indel loci. When different-sized fragments were present in at least one of the five species, the indel-containing region was sequenced (Table 2).

**Table 2:** Laboratory results of the insertions/deletions that are predicted to have an effect on coding regions of genes

**Gene**	**Size (bp)**	**Impact**	**Confirmation**	**Ancestral state**
*NEFH*	24	truncation from 1020 to 753 aas	yes	unknown
*CELSR1*	21	7 aa insert	unknown	-
*FLJ44385*	68	truncation from 125 to 113 aas	yes	Del
*UNC84B*	65	truncated from 717 to 68 aas	yes	Del
*TCF20*	515	truncation from 1960 to 1893 aas	unknown	-
*SMC1L2*	74	truncates from 1235 to 1220 aas	yes	Ins
*FLJ41993*	36	undetermined	yes	unknown
*RUTBC3*	250	truncates from 849 to 734 aas	different ins	unknown
*NEFH*	18	truncated from 1020 to 252 aas	unknown	-
*RUTBC3*	13	substitution in protein	unknown	-
Various*	10–148	splice site mismatch with no impact		

With the use of GENSCAN, the 23 indels found in coding exons or in splice site regions of the human REFseq genes were analyzed for their impact to these genes. Out of the 23, 10 of these indels impact genes through truncation/insertion of amino acids or substitution within a protein. The other 13 indels had no affect on the proteins generated from genes *DGCR8, ZDHHC8, BRD1, SELO, SBF1, CHKB, PCQAP, BCR, PRODH, RUTBC3, CYP2D7P1*, &*ACSIN2*. In order to confirm the presence of an insertion or a deletion, the loci and its harboring regions were sequenced in each of the species. Assembly confirmation is dependent on whether the indel product is completely present (all of the bases) in the corresponding species predicted. For gene *FLJ44385*, the indel was present in its entirety. The assembly was also confirmed for genes *SMC1L2 *and *FLJ41993 *with all of the bases of the indel present, however additional sequence was also detected. The inferred ancestral state of each indel is based on whether a species has the insertion or deletion of that locus and the species' position on the phylogenetic tree.

* *DGCR8*, *ACSIN2*, *BCR*, *BRD1*, *CHKB*, *CYP2D7P1*, *DGCR8*, *PCQAP*, *PRODH*, *RUTBC3*, *SBF1*, *SELO*, &*ZDHHC8*

**Indel verified and additional sequence is present

The results of the laboratory validation varied among indels. For instance, in the *FLJ44385 *gene, the human insertion was present in its entirety and led to the truncation of the protein from 125 to 113 amino acids (Table 2). Predicted indels were found and their impacts confirmed for genes *SMC1L2 *and *FLJ41993 *also. However, in *FLJ41993 *an additional sequence that is not present in the reference sequence (Build 36) was also detected. The *UNC84B *sequencing yielded an incomplete fragment of the predicted indel. One of the sites in the *RUTBC3 *genes was amplified, but with an indel different from the human draft sequence. DNA amplified from another site within *RUTBC3 *and one in *TCF20 *did not show any detectable differences in PCR fragment size in the different species. Computationally predicted indels within *NEFH *were additionally corroborated by human traces alignments (trace IDs: 1181046117, 1183837089, 1200564777, 1227912385, 1229518898, and 546289986). In the experimental validation, only one of the sites at *NEFH *was successfully amplified with the indel size and sequence as predicted. The regions containing other indel site at *NEFH *and indel within *CELSR1 *did not amplify, despite numerous attempts.

## Discussion

Indels that differentiate between humans and chimpanzees likely contribute to the speciation process [23,26] of these closely related species. In the current analysis, a comprehensive evaluation of insertion/deletion variations between human and chimpanzee chromosome 22 was performed. Upon examination, 1,429 out of 6,278 indels were found in genomic areas free of known repetitive DNA and STRs on chromosome 22 (Table 1), and these were examined with respect to their structure, length, and location relative to known structural gene elements, and also the number, sequence similarity, and the location of the additional copies. We compared observed distributions of different indel classes to the baseline distribution of an expected set generated by a tenfold resampling approach (see Materials and Methods), as well as among one another. We also developed a framework for analysis of local repetitive structure around indels. Our results support the observation that indels have had a key role in primate evolution and contribute to important genetic differences between humans and chimpanzees [10].

Many indels from the syntenic human and chimpanzee chromosome 22 sequences were located around known repetitive DNA, and initially filtered out (4,849 out of 6,278). The remaining 1,429 were distributed unevenly across the genome features (Fig. 3). Indels were found under-represented within functional elements (Fig. 3), probably due to some selective constraints. This situation is not unique to indels as a subtype among other genomic variants. Previously, studies have shown that indel events and single nucleotide substitutions correlate, and there may be common underlying properties for both processes, since indels accumulate in the same parts of the genome that exhibit higher substitution rates [19] with hotspots often coinciding [10]. As is the case in single substitutions, indels occur with different frequencies across the genome. Specifically, indels are scarcer and shorter in coding sequences [19] where selective constraints are the most intensive, and 3 bp indels in the coding regions, which maintain the coding sequence without frameshifts, are by far the most common class [22]. In the current study, the approximate class of indels is on average larger and located the furthest from the coding sequences (Fig. S3(see Additional file 1)). Approximate indels were only half as numerous as expected within genes (6.5% vs. 13%, Table S4), and within the functional elements, they were both shorter than, and not as numerous as the unique and exact ones (Table S2.1A (see Additional file 1)). In addition, approximate indels were furthest away from the nearest exons (Table S2.1B (see Additional file 1)).

Only 6.5% of all indels characterized in our study were parts of gene elements (positioned within promoters, splice and terminator sites, untranslated and coding regions of known genes), nearly a half as many as expected in comparison to the baseline represented by the resampled data (Fig. 3, bottom panel). Only in the 3' gene flanking regions did indels occur at frequencies similar to the expectation in the resampled dataset. The presence of selective constraints acting on functional elements of the genome probably explains why the intergenic regions contain many more indels than genes [22]. However, somewhet unexpectedly, indels in the introns were more frequent than in the intergenic regions (Fig. 3). The paucity of intergenic indels is consistent with the presence of unknown regulatory elements or genes [27]. Other studies point out to a twofold larger number of intronic over the intergenic indels [22], but the filtering of repetitive DNAs and the 10-bp cutoff applied in this study likely accounts for the reduced number of intergenic indels observed. Our estimate of the number of indels within five gene elements (6.5%) and introns (41.8%), if combined, is higher than previously reported (35.7% [10]). This may reflect our choice to focus on indels longer than 10 bp.

### Structural characteristics and origins of indels

Together with identification of the sequence context around indels, the structural characteristics of local repeats proved to be useful for systematic understanding of their nature and origin. More recently, non-homologous end joining mechanism, one of the consequences of double-strand breakage [15], and non-allelic homologous recombination have been proposed as main indel-generating mechanisms [24,28]. Still, the role of repeats as the underlying mechanism that gives rise to insertions and deletions should not be overlooked, especially given the indel proximity to known repetitive DNA [7,20,29], and their frequent association with short duplications [23,30,31]. Other work suggested that indels have been generated mainly by unequal crossing over and replication slippage [22].

All of these molecular mechanisms generate copies of indels, making the presence of identical sequences locally, on the chromosome, and genome-wide a point of interest (Fig. 1 and S1 (see Additional File 1)). We followed this scheme and found that most of the observed indels in this study (1,317 of 1,427) did not have any exact or approximate copies anywhere else in the genome (genome-unique, Fig. 2, Table S1, and Fig. S2 (see Additional file 1)), but 103 of them had copies present on the same chromosome (chromosome-unique), and seven were found on other chromosomes (chromosome-multiple, Fig. 2 and Fig. S2 (see Additional file 1)).

Current analysis focused on delimiting three core indel classes (Fig. 1), based on exact or approximate copies of the indel in the flanking sequence (± 5 Kb), or its unique presence (see Materials and Methods and Fig. S4 (see Additional file 1)). Differences in repeat structure among indels suggested that a local mechanism of indel formation may be the result of two main duplication processes, where new local duplications, resulting in the exact indel core class, and the subsequent nucleotide substitution generate indels in the approximate class. The origins of the unique core class are not clear, but could be partially explained by a smaller-than-10-bp local repeat structure (our repeat finder analysis size). Approximate and exact copy indels were over-represented, while unique indels were under-represented in the observed dataset (Fig. 2). While numerous elsewhere, approximate indels were scarce within genes, and especially within coding sequences, possibly a consequence either of negative selection, and/or their older evolutionary age, compared to the members of other core classes (Fig. 2). Age of indels may also account for other observations in this study: while exact indels are longer than expected, approximate indels are both shorter and located further away from genes than expected (Fig. 4, Table S2.1, and Fig. S3 (see Additional file 1)). Unfortunately, the exact core class of indels was not well represented in our survey (168 observed and 141 resampled indels, Fig. 2 and Fig. S2 (see Additional file 1)), and making inferences about this important class was more tentative.

Since distances between conserved duplications are highly preserved between humans and chimpanzees [30], we were able to search for numbers of repeats of each indel sequence among the indel classes. Most of the approximate and unique indels were found in clusters represented by four or more copies. These included more than half of exact indels (88 of 168) and more than 2/3 of the approximate ones (586 of 831). This suggests that the number of nearby copies in the two respective classes is an important characteristic of indels, and has a potential to serve as a metric of the evolution of duplicates.

### The scale and validation of indel impacts

Insertions and deletions comprise less than one percent of known disease-causing mutations [23]. Some indels can have a significant effect on protein product and gene expression, especially when they are located within functional gene elements [10]. According to some estimates, at least 13% of the genes harbour at least one indel [19]. Insertions in coding sequences often result in translational frameshifts, with the consequence of premature protein termination or nonsense-mediated RNA decay [19]. It is highly likely that some of them are actually disease-causing, and several indels have already been included in a list of candidate genes for cancer and other disorders [32-35]. Our approach identified 23 indels in coding exons or in splice site regions of human RefSeq genes (Fig. 5). Among these, 10 indels were found to alter the sequence of a corresponding protein, and our experimental validation confirmed five: *FLJ44385*, *FLJ41993, NEFH*, *SMC1L2*, and *UNC84B *(Table 2).

A parallel study of genetic variation on human chromosome 22 [36] suggested that humans harbour similar levels of indel variation: indels represented 22% of the total polymorphic events, which is somewhat more than the 18% reported earlier, but within the predicted range estimate of 16% to 25% of all sequence polymorphisms in humans [10]. Our findings support the conclusion that a large fraction of the differences between human and chimpanzee genomes is comprised of indels [22]. However, not all indels have been validated, or even sequenced; therefore, those estimates should be cautiously evaluated. Only three of the 10 indels in the coding sequence that had a potential impact on gene function revealed the same indel after sequence analysis (Table 3).

One of the limitations of the present work was that it utilized the low coverage draft sequence of the chimpanzee genome and relatively early version of the human genomic sequence. However, even an unfinished chimpanzee genomic sequence could be useful for detection of the gene-modifying indels, as demonstrated by previous work [37]. Our computational and experimental data partially support this notion. Unfortunately, incomplete data that consists of misassembled or poor-quality sequences, and unsequenced genomic fragments can also contribute to the high volume of false-positive indels. We addressed this problem in the follow-up validation by PCR and sequencing. Not surprisingly, we encountered primate sequences different from anything currently listed in public databases (Table 2). In the future, higher coverage genomic sequences of the different primate species should allow a more accurate identification of the loci that differentiate primates closely related to humans [38].

## Conclusion

In the current report, we investigated and discussed several aspects of the indels phenomenon: (i) indels represent the abundant group of the genomic sequence variations; (ii) the mechanism of their formation is potentially reflected by the local structure of the indel containing region; (iii) indels represent a source of the potential mutations in primate genes and genomes; (iv) indels can play an important role in the understanding of genome evolution and genome plasticity; and (v) indels are an important of source polymorphisms that directly influence human phenotypes and diseases.

## Methods

### Sequence alignment and identification of indels

Syntenic regions of human chromosome 22 (Genome Assembly July 2003, hg16) from 15.4 to 49.3 Mb and chimpanzee chromosome 22 Chimpanzee (Genome Assembly Dec 2003) from 15.4 to 50.0 Mb were retrieved from the UCSC database and Ensembl database, respectively. Coordinates are from the August 2003 release of the human sequence and Ensembl May 2004 of the chimpanzee. Syntenic fragments (33 Mb) from human chromosome 22 and chimpanzee chromosome 22 (Human Genome Assembly [July 2003]) and Chimpanzee Genome Assembly (Dec 2003) were aligned using the MUMmer program [39,40]. MUMmer output was parsed to identify small insertions and deletions (with lengths of 10 bp to 10 Kbp). The evolutionarily neutral term "indel" was used to signify these length differences. All indel sequences surrounded by at least 10 bp of perfectly aligned flank and with no more than 50% undetermined bases (Ns) were selected for further analysis (Table 1). All coordinates were transferred to the most recent genome assembly using LiftOver  for reference and given in the Supplementary Materials (Table S6 (see Additional file 2).

### Characterization of indel sequences

The insertion sequences with 500 bp of flanking sequence were extracted. The selected fragments were analyzed with RepeatMasker and Tandem Repeat Finder [41]. Sequences that overlapped with known repetitive elements and/or short tandem repeats (≥ 10 bp) were filtered from the original data set. The remaining indel data set was subjected to a more detailed analysis of the local and genomic features. Insertion sequences were extracted from human and chimpanzee chromosomes, and BLAT [42] searches were performed with these fragments against the entire human genome assembly to identify genomic locations harbouring identical sequences (± flanking 18 bp). The local repeat structure of the indel region was examined in the 5 Kbp of flanking genomic regions on both sides (Fig. S4 (see Additional file 1)). New repeats and repeat classes in these regions were found using REPuter [43,44] and RepeatFinder [45], utilizing a minimal repeat length of 10 bp and a gap of 1 bp for repeat clustering parameters. With these results, indels were categorized into the classes based upon the local structure of the indel region (Fig. 1).

### Resampling and statistical analysis of indel data

We generated a random data set to examine the significance of the identified indels' features and classes. The random set of sequences was created using the following rules: (i) human genomic sequence was selected as the source of all sequences; (ii) coordinates of beginnings of randomly chosen sequences were selected from the range and frequency distribution of the analyzed human chromosomal fragments; (iii) the number of generated resampled sequences was 10 times larger than the number of indels in the original data set (Fig. S1 (see Additional file 1)). This random set of sequences was filtered using the same criteria as the original set of observed indels where N-rich sequences, known repeats, and tandem repeats were deleted. Then, a subset of this cleaned data was extracted so that the distribution of the sequence lengths matched exactly the distribution of sequence lengths in the original or observed indel set (Fig. S1 (see Additional file 1)). Finally, the local structures of the indel regions in the resampled data were determined using the same procedures as with the observed indels. These procedures are described in the next section. Statistical significance of the described classifications was assessed by linear general models regression procedures (GLM). Fisher exact test was applied for the table analysis. All statistical analyses were performed with SAS Version 9.1 software (SAS Inc., Carey, NC)[46].

### Location of indels relative to known genes

Coordinates of the exons and coding regions of the REFseq genes annotated on human chromosome 22 (553 transcripts) were downloaded from the UCSC genomic database. We measured distances between each indel and the nearest exon, represented by the distance between the closest ends of both features. Next, we classified the entire dataset based on the distance of the indel to the nearest exon, the indel and exon lengths, the strand of the exon, exon number, and the coding region coordinates. Thus, we defined 10 general gene-related groups of the indels: (i) intergenic – the shortest distance from the indel to the nearest first and/or last exons of the neighbouring genes was greater than 25 Kb; (ii) upstream – the indel was 2–25 Kb away from the 5' side of the first annotated exon of a gene; (iii) downstream – the indel was 2–25 Kb away from the 3' side of the last exon of a gene; (iv) promoter – the indel was positioned less than 2 Kb away from the 5' side of the annotated first exon; (v) terminator – the indel was sited less than 2 Kb from the 3' side of the last exon; (vi) intronic – the indel was located in its entirety between the exons of the same gene; (vii) 5' UTR – the indel was found within an exon but outside the coding region on the 5' side; (viii) 3'UTR – the indel was found within an exon but outside the coding region on the 3' side; (ix) exonic – indel was contained entirely within an exon and within its coding region; (x) splice-site – the indel coordinates overlapped either the beginning or the end of an exon.

### Estimation of indel impact on gene structure and protein product

We concentrated on those indels with the highest potential impact on their protein product, and subsequently, on the biological function. Hence, using Build 36 Genome Assembly (February 2006, hg18), 23 indels found either in the coding sequences of exons or in the splice site regions of human REFseq genes were analyzed for their possible impact on those genes. For each gene we collected 2 sequences: first, a fragment of human chromosome 22, including the entire gene transcript; second, a putative sequence constructed from the first fragment by suppressing the indel, either by deleting the insertion or by adding the predicted deletion sequence of chimpanzee origin. Both sequences were analyzed using GENSCAN [47]. Differences between protein products predicted in these pairs were reported to characterize the indel impact.

### Primer design

A semi-automatic procedure was developed to identify primers in the indel regions. The procedure identified conserved regions in the flanks of the selected indel by parsing the MUMmer alignment, then pairs of the primers were predicted by the primer3 [48] and the uniqueness of the primer pairs in both human and chimpanzee genomes was checked. The uniqueness rule was implemented as follows: each pair of primers was mapped by BLAT [42] to the human and chimpanzee genomic sequences. One of the primer sequences had to be unique in each genome and the other one could not have more than 10 copies per genome to be selected as a unique PCR set. Some indel regions did not satisfy such criterion, so, manual choosing was performed.

### DNA samples

We selected 189 DNA samples from five species of great apes, as well as several populations of humans. The apes in the sample set included 26 chimpanzee (*Pan troglodytes*), 11 bonobos (*Pan paniscus*), and nine gorillas (*Gorilla gorilla*). The set also included two orangutan subspecies: five samples of Sumatran (*Pongo pigmaeus abelii*) and five samples of Bornean orangutans (*P. p. pigmaeus*). Most of these were available from within the Laboratory of Genomic Diversity (LGD) repository. The remainder was purchased from either the Integrated Primate Biomaterials and Information Resource (IPBIR, beginning with PR) or the Coriell Institute for Medical Research, Camden, NJ (beginning with NG and NS). Purchased chimpanzee samples were PR00226, PR00496, PR00512, PR0064, PR00744, NS03629, NS06939, NS03487, NS03639, NS03641, NS03659, NS03656, NS03650, NS03660, NS03623, NS03622, NS03657, NS0361; bonobo (PR00092, PR00251, PR00367, PR00661, PR00446, PR00111, NG05253); and gorilla (NG05251, PR00107, PR00573). To our knowledge, all of these were unrelated individuals, except for one father-son pair of gorillas from the LGD repository (GGO-3 and GGO-7). The human set of samples represented five distinct ethnic groups: Asians (n = 24), European Americans (n = 25), Amerindians (n = 25), African Americans (n = 25), Senegalese (n = 25), and Botswanian (n = 9). These DNA samples were some of the same samples we have characterized previously [49].

### Indel verification

Twenty-three indels were selected for experimental evaluation and validation based on their predicted gene impacts. At each locus, five nanograms of human or primate genomic DNA were amplified with *AmpliTaq *gold (Applied Biosystems) and the touchdown PCR protocol was used with the Applied Biosystems 9700 thermal cyclers. The touchdown protocol conditions were the following: we started reactions with 9 min heating at 94°C, followed by 5 cycles of 30 sec at 94°C, 30 sec at 65°C, and 30 sec at 72°C, subsequently followed by 21 cycles at the same conditions, except lowering the annealing temperature by 0.5°C at each cycle (to 55°C), continued by 15 cycles of 30 sec annealing at 94°C, 30 sec at 55°C, and 30 sec at 72°C, and finished by 10 min of final extension at 72°C. Indels were initially detected by PCR amplification, analyzed on 4% agarose gels using 0.5× TBE buffer. Gels were run for three hours at 100 volts and stained with 0.5 ug/mL EtBr for 30 min. PCR primer sets were tested on all 189 samples.

A subset of 12 human and 12 primate samples was selected for sequencing those fragments that exhibited indel polymorphisms in the larger panel. The subset included: European (n = 3), Asian (n = 3), American Indian (n = 3), Senegalese (n = 3), African American (n = 3), Botswanian (n = 3), gorilla (n = 4), chimpanzee (n = 3), bonobo (n = 3), Sumatran (n = 2), and Borneo orangutan (n = 2) samples. PCR products from the subset were sequenced by using ABI BigDye Terminator and ABI PRISM 3730 DNA Analyzer (Applied Biosystems, Foster City, CA). Sequence results were analyzed with Sequencher 4.2 software. Each experimental sequence was checked against the public datasets and reanalyzed as described above for indels, because some were different from the genomic sequences in the databases.

## Authors' contributions

NV, TKO, RMS and MWS designed the experiments. NV, TKO and RMS performed computational and statistical analysis. KCC performed PCR reactions and sequencing. ALT obtained and managed samples, helped with sequencing protocols. All authors contributed to the writing of the paper. NV, TKO, and MWS wrote the final manuscript.

## Supplementary Material

Additional file 1 **Supplemental Tables and Figures.** This file contains the following supplemental tables and figures: Table S1. Nonrandom distribution of genome classes among the core classes; Table S2.1A1. Indel length distribution among gene elements and core classes; Table S2.1A2. Indel length distribution among gene elements; Table S2.1B. Distance to the closest exon among gene elements and core classes; Table S2.1B2. Distance to the nearest exon distribution among gene elements; Table S3. Differences of the observed vs. resampled distribution of core classes of indels in the locations with respect to gene elements; Table S4. Differences of the observed vs. resampled distribution of indels in the locations with respect to gene elements; Table S5. Differences between insertions and deletions in genetic and core categories; Figure S1. Length distribution of the observed and resampled indel in the three indel classes; Figure S2. Distribution of core classes among the genome classes of indels; Figure S3. Distance from the indel to the nearest exon; Figure S4. Identification of unique indels. Click here for file

Additional file 2 **Supplemental Tables S6**. This file contais complete information on coordinates and cahracteristics of indels used in this study. Click here for file
