# Supplementary material for: Genome and gene alterations by insertions and deletions in the evolution of human and chimpanzee chromosome 22
Source: BMC Genomics. 2009 Jan 26;10:51. doi: 10.1186/1471-2164-10-51 (PMC2654908; doi:10.1186/1471-2164-10-51)
Supplement: Additional file 1 — Supplemental Tables and Figures. This file contains the following supplemental tables and figures: Table S1. Nonrandom distribution of genome classes among the core classes; Table S2.1A1. Indel length distribution among gene elements and core classes; Table S2.1A2. Indel length distribution among gene elements; Table S2.1B. Distance to the closest exon among gene elements and core classes; Table S2.1B2. Distance to the nearest exon distribution among gene elements; Table S3. Differences of the observed vs. resampled distribution of core classes of indels in the locations with respect to gene elements; Table S4. Differences of the observed vs. resampled distribution of indels in the locations with respect to gene elements; Table S5. Differences between insertions and deletions in genetic and core categories; Figure S1. Length distribution of the observed and resampled indel in the three indel classes; Figure S2. Distribution of core classes among the genome classes of indels; Figure S3. Distance from the indel to the nearest exon; Figure S4. Identification of unique indels. [file 1471-2164-10-51-S1.pdf]

Supplemental materials for:

Genome and gene alterations by insertions and deletions in the evolution of human and chimpanzee chromosome 22

Natalia Volfovsky[1#], Taras K Oleksyk[2,3,4#], Kristine C Cruz[2], Ann L Truelove[2,3], Robert M Stephens[1], and Michael W Smith[2,3§]

[1]Advanced Biomedical Computing Center, Advanced Technology Program, SAIC-Frederick, National Cancer Institute at Frederick, Frederick, MD 21702
[2]Laboratory of Genomic Diversity, National Cancer Institute at Frederick, Frederick, MD 21702
[3]Basic Research Program, SAIC-Frederick, National Cancer Institute at Frederick, Frederick, MD 21702
[4]Department of Biology, University of Puerto Rico, Mayagüez, PR 00681
[§]Corresponding author
[#] Contributed equally

E-mail addresses:
NV: natalia@ncifcrf.gov
TKO: oleksyk@ncifcrf.gov
KCC: cruzkc@gmail.com
ALT: anntruelove@gmail.com
RMS: bobs@ncifcrf.gov
MWS: smithm@ncifcrf.gov

Address correspondence to:

Michael W. Smith
SAIC-Frederick
National Cancer Institute at Frederick
Bldg 560, Rm 21-74, Frederick, MD 21702 USA
Phone: 301 846-1913
Fax: 301 846-1909
E-mail: smithm@ncifcrf.gov

Supplemental Tables

Table S1. Nonrandom distribution of genome classes among the core classes (LR χ^2, d.f.=4, χ^2=23.28, p=.0001).

	Approximate n(%)	Exact n(%)	Unique n(%)	Total
Multiple on chromosome 22 (CM)	2(28.6)	0(0)	5(71.4)	7
Unique to chromosome 22 (CU)	78(75.7)	10(9.7)	15(14.6)	103
Unique on the genome (GU)	741(56.3)	158(12)	418(31.7)	1317
Total	821	168	438	1427

Table S2.1A1. Indel length distribution among gene elements and core classes.

Indel Core Class		Downstream	Functional Elements	Intron	Upstream	Intergenic	Total	Among Core Groups
Approximate	**length (obs)**	43.8 (15.68-122.39)	40.01(15.09-106.04)	47.23(18.42-121.11)	50(17.4-143.66)	48.48(18.78-125.12)	47.16(44.17-50.35)	-"-
	**length (exp)**	50.76(18.18-141.68)	64.05(20.68-198.35)	50.18(18.64-135.11)	51.6(18.93-140.65)	51.38(19.21-137.43)	52.32(51.14-53.53)	
	β	0.06	0.20	0.03	0.01	0.03	0.05	0.49
	p	0.142	**<.0001**	0.175	0.729	0.245	**0.0003**	**<.0001**
Exact	**length (obs)**	17.94(13.36-24.09)	18.74(13.41-26.18)	19.2(11.04-33.42)	19.05(13.14-27.6)	18.87(11.46-31.08)	18.99(16.43-21.94)	-"-
	**length (exp)**	19.75(9.12-42.77)	13.34(8.06-22.07)	15.85(8.4-29.9)	15.09(9.35-24.36)	17.1(10.15-28.82)	16.19(13.84-18.93)	
	β	0.04	-0.15	-0.08	-0.1	-0.04	-0.07	0.08
	p	0.799	0.321	0.138	0.43	0.541	0.07	0.156
Unique	**length (obs)**	13.01(10.11-16.75)	16.11(11.49-22.58)	15.76(11.03-22.51)	17.74(10.48-30.04)	15.6(10.56-23.04)	15.67(14.33-17.14)	-"-
	**length (exp)**	18.45(11.38-29.9)	18.51(11.45-29.94)	18.48(11.38-30.03)	18.52(11.39-30.12)	18.62(11.58-29.93)	18.53(18.13-18.93)	
	β	0.15	0.06	0.07	0.02	0.08	0.07	*reference group*
	p	**0.01**	0.266	**0.009**	0.732	**0.007**	**<.0001**	

Indel lengths distributions in each of the gene elements in each core class are compared to their resampled distributions, and the difference (*β*) and significance (*p*) of each comparison in listed. Length distributions of approximate and exact indels are compared to the unique indels. The significance of the results here and elsewhere in the paper was adjusted in the general model using multivariate regression analysis. There was a significant difference in the observed length distribution among the lengths of indels within gene elements and core classes and their resampled values (GLM, $p<0.0001$).

Table S2.1A2. Indel length distribution among gene elements.

Gene elements			Compared to the resampled		Compared to the intergenic	
	**length (obs)**	**length (exp)**	β_{oe}	p(t)	β_{al}	p(t)
**Downstream**	27.57(22.81-33.33)	30.44(28.71-32.28)	1.1	0.283	-0.08	0.217
**Functional Elements**	25.83(21.22-31.45)	34.74(33.09-36.47)	1.35	**0.001**	0.01	0.817
**Intron**	30.32(27.93-32.92)	29.21(28.45-29.99)	0.96	0.351	0.004	0.907
**Upstream**	33.46(28.16-39.75)	29.9(28.29-31.6)	0.89	0.18	0.06	0.350
**Intergenic**	30.88(28.13-33.89)	29.57(28.68-30.49)	0.96	0.343		*reference*

Indel length in each of the gene elements is compared to the resampled distribution and to the and to the distribution of indel length in the intergenic sequence (reference).

Table S2.1B. Distance to the closest exon among gene elements and core classes.

Indel Core Groups		Downstream	Functional Elements	Intron	Upstream	Intergenic	Total	Among Core Groups
Approximate	**distance (obs)**	8975(4393-18336)	341(86-1353)	1563(231-10553)	10176(5030-20586)	137790(53564-354453)	8886(7865-10039)	-"-
	**distance (exp)**	8652(4309-17374)	146(22-957)	1547(212-11303)	8708(4258-17811)	108968(45321-261995)	5410(5173-5657)	
	β	-0.02	-0.37	0.003	-0.07	-0.1	-0.216	0.15
	***p*(t)**	0.852	**<.0001**	0.906	0.381	**0.017**	**<.0001**	**0.023**
Exact	**distance (obs)**	10661(4989-22781)	458(111-1891)	1584(228-11007)	8563(4444-16500)	118293(42389-330118)	5792(4423-7585)	-"-
	**distance (exp)**	11453(7186-18254)	314(59-1677)	1639(336-7984)	6316(2674-14916)	89868(32695-247017)	7540(5543-10255)	
	β	0.03	-0.16	0.01	-0.13	-0.12	0.115	0.08
	p	0.922	0.573	0.893	0.597	0.384	0.3871	0.459
Unique	**distance (obs)**	10023(5339-18816)	387(87-1718)	1175(156-8861)	8367(4053-17273)	98306(41047-235439)	6877(5820-8127)	-"-
	**distance (exp)**	8676(4357-17274)	165(23-1194)	1651(236-11547)	8702(4399-17216)	111189(45435-272098)	6188(5931-6455)	
	β	-0.06	-0.37	0.15	0.02	0.05	-0.046	*reference*
	p	0.585	**0.001**	0.004	0.872	0.336	0.4207	

Distance to exon distributions in each of the gene elements in each core class are compared to their resampled distributions, and the difference (*β*) and significance (*p*) of each comparison in listed. Distance to the closest exon distributions of approximate and exact indels are compared to the unique indels. The significance of the results here and elsewhere in the paper was adjusted in the general model using multivariate regression analysis. There was a significant difference in the observed distance to exon distribution among the of indels within different gene elements and core classes and their resampled values (GLM, p<0.0001).

Table S2.1B2. Distance to the nearest exon distribution among gene elements.

Gene elements			Compared to the resampled		Compared to the intergenic	
	**distance (obs)**	**distance (exp)**	β_{oe}	***p*(t)**	β_{al}	***p*(t)**
**Upstream**	9444(6630-13453)	8679(7736-9736)	0.92	0.572	-0.99	**<.0001**
**Functional Elements**	370(256-534)	156(141-171)	0.42	**<.0001**	-2.41	**<.0001**
**Introns**	1444(1239-1683)	1602(1522-1687)	1.11	0.11	-1.92	**<.0001**
**Downstream**	9441(6847-13019)	8673(7779-9670)	0.92	0.534	-1.07	**<.0001**
**Intergenic**	121927(102490-145054)	109901(103505-116693)	0.9	0.16	*reference*	

Distance to the nearest exon within groups of each of the gene elements is compared to the resampled distribution and to the and to the distribution of distance to the nearest exon in the intergenic sequence (reference).

Table S3. Differences of the observed vs. resampled distribution of core classes of indels in the locations with respect to gene elements

Core Classes	Among Location Categoiries† (d.f.=4)		In Genes (d.f.=1)		In CDS (d.f.=1)	
	χ^2	**p-value**	χ^2	**p-value**	χ^2	**p-value**
Approximate	31.60	**<.0001**	6.19	**0.01**	8.84	**0.003**
Exact	4.30	0.37	3.02	0.08	2.40	0.12
Unique	3.33	0.50	2.55	0.11	12.73	**0.0004**
Overall	24.72	**0.02**	1.03	0.60	12.51	**0.0012**

† Location categories include: intergenic, upstream, intron, in-genes and downstream

Table S4. Differences of the observed vs. resampled distribution of indels in the locations with respect to gene elements

	Approximate			Exact			Unique		
Locations	Observed (%)	Resampled (%)	p(χ^2)	Observed (%)	Resampled (%)	p(χ^2)	Observed (%)	Resampled (%)	p(χ^2)
downstream	67(8.16)	578(8.65)	*0.86*	11(6.55)	7(4.96)	*0.47*	35(7.99)	584(7.85)	*0.96*
in genes	53(6.46)	872(13.05)	*0.05*	12(7.14)	9(6.38)	*0.76*	41(9.36)	826(11.11)	*0.58*
in introns	343(41.78)	2,659(39.78)	*0.68*	88(52.38)	61(43.26)	*0.07*	171(39.04)	3,065(41.22)	*0.66*
upstream	83(10.11)	613(9.17)	*0.75*	13(7.74)	15(10.64)	*0.35*	41(9.36)	677(9.1)	*0.93*
intergenic	275(33.5)	1,962(29.35)	*0.36*	44(26.19)	49(34.75)	*0.07*	150(34.25)	2,284(30.72)	*0.44*
obs vs exp across all the locations	821	6,684	*0.39*	168	141	*0.39*	438	7,436	*0.94*

Table S5. Differences between insertions and deletions in genetic and core categories

	length (F=14.42, *p=.0002*)*			distance to exon (ns)		
	β	*t*	*p*	*β*	*t*	*p*
**Genome Locations**						
downstream	-0.04	-0.35	*0.72*	0.15	0.68	*0.50*
in genes	0.04	0.39	*0.70*	-0.06	-0.32	*0.75*
in introns	0.04	0.82	*0.41*	0.04	0.36	*0.72*
upstream	0.13	1.17	*0.24*	0.21	1.00	*0.32*
intergenic	0.04	0.69	*0.49*	0.09	0.82	*0.41*
**Core categories**						
approximate	0.10	2.54	***0.011***	0.02	0.30	*0.76*
exact	-0.02	-0.23	*0.82*	-0.11	-0.54	*0.59*
unique	0.04	0.69	*0.49*	0.09	0.82	*0.41*

Supplemental Figures

Figure Legends

Figure S1. Length distribution of the observed and resampled indel in the three indel classes.

The random resampled set of sequences was created using human genomic sequence where coordinates of randomly chosen sequences were selected from the range of analyzed human chromosomal fragments. This resulted in two distributions (observed and resampled) with exactly the same shape, but there are 10x more indels in the resampled category.

Figure S2. Distribution of core classes among the genome classes of indels.

First, we the observed into three genome classes according to the presence and the location of additional copies of the indel sequences in the human genome. Indels whose exact sequence (± 18bp) was unique to their location were designated as"unique to the genome", 92.3%, followed by the indels with copies present only on chromosome 22 ("unique to chromosome 22", 7.2%), and the reminder had copies found elsewhere in the genome ("multiple times on chromosome 22", 0.5%). In addition, three major core types were described based on local structure analysis: (i) those found uniquely in the 10Kb indel harbouring region were marked "unique", (ii) those with at least one exact copy of the indel sequence found locally were categorized as "exact", and (iii) those with similar but not identical copies found locally were regarded as "approximate" (Fig 1). There seems to be more approximate indels unique to chromosome 22 (see Results).

Figure S3. Distance from the indel to the nearest exon.

(A) Frequency distributions of distances between the indels and the nearest exons among the three core classes of the indels. (B) Comparative distributions among observed (solid colors) and resampled (line) distances from the indels to the closest exon (%). Approximate indels in the observed dataset (n=821) were further from the exons than resampled (Table S2.1B, $p<0.0001$).

Figure S4. Identification of unique indels.

Those indels that has no copies of its original sequence (±18bp) in the 10Kb indel harbouring region were marked "unique"

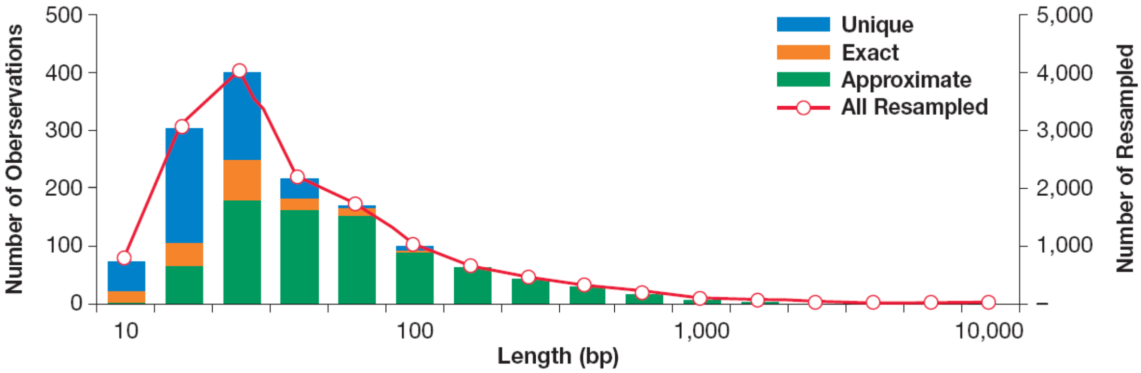
Unique
Exact
Approximate
All Resampled
Number of Oberservations
500
400
300
200
100
0
10
100
1,000
10,000
Length (bp)
5,000
4,000
3,000
2,000
1,000
–
Number of Resampled

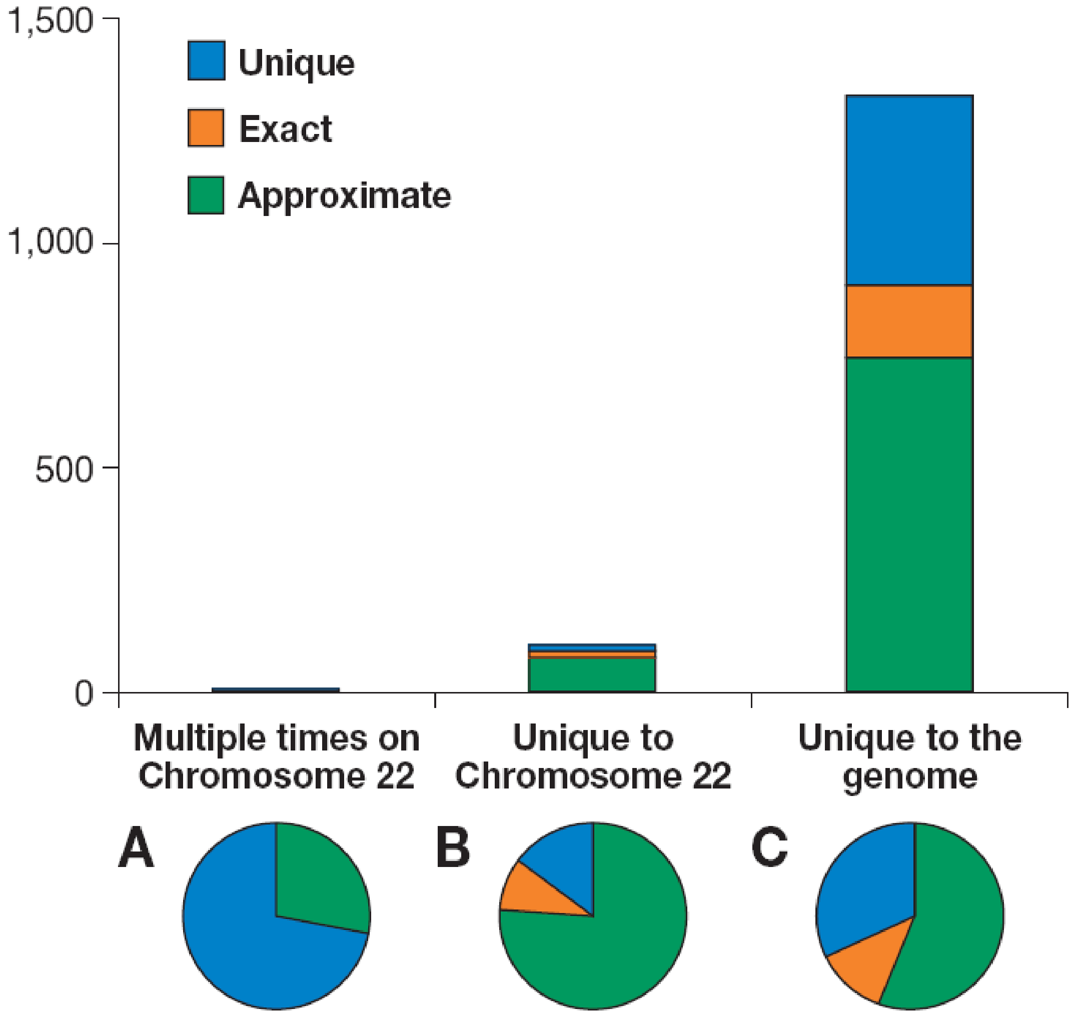
1,500
1,000
500
0
Unique
Exact
Approximate
Multiple times on Chromosome 22
Unique to Chromosome 22
Unique to the genome
A
B
C

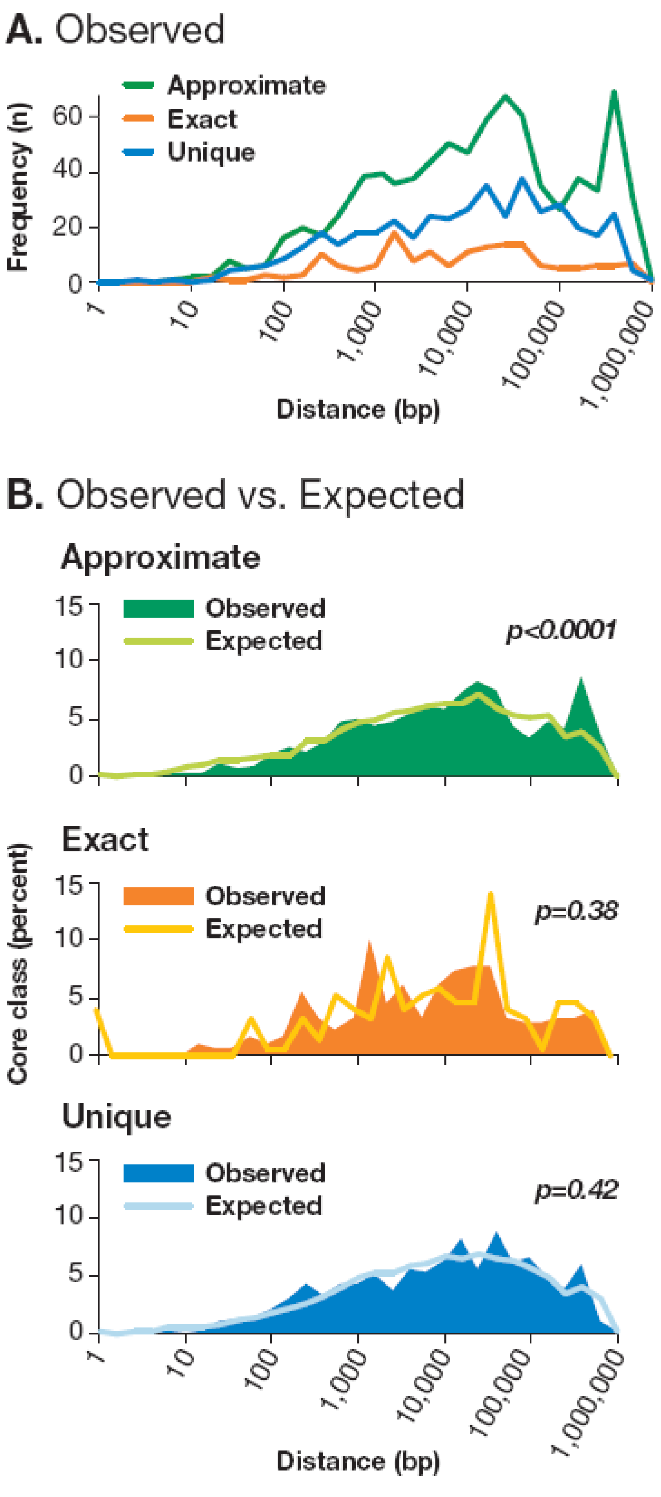
A. Observed
Approximate
Exact
Unique
Frequency (n)
60
40
20
0
1
10
100
1,000
10,000
100,000
1,000,000
Distance (bp)
B. Observed vs. Expected
Approximate
Observed
Expected
p<0.0001
Exact
Observed
Expected
p=0.38
Unique
Observed
Expected
p=0.42
Core class (percent)
15
10
5
0
Distance (bp)

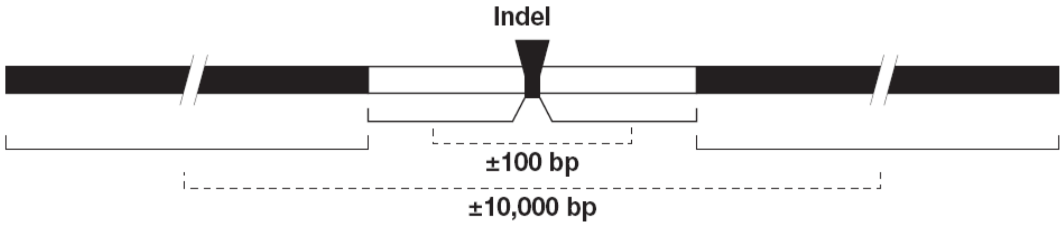
Indel
±100 bp
±10,000 bp
